# Continuous Electrochemical Carbon Capture via Redox-Mediated pH Swing—Experimental Performance and Process Modeling

**DOI:** 10.1021/acs.jpclett.4c03111

**Published:** 2025-01-29

**Authors:** P. Śledzik, P.M. Biesheuvel, Q. Shu, H.V.M. Hamelers, S. Porada

**Affiliations:** † Department of Process Engineering and Technology of Polymer and Carbon Materials, Wroclaw University of Science and Technology, Wyb. St. Wyspiańskiego 27, 50-370 Wrocław, Poland; ‡ Wetsus, European Centre of Excellence for Sustainable Water Technology, Oostergoweg 9, 8911 MA Leeuwarden, The Netherlands; § Environmental Technology, Wageningen University, Bornse Weilanden 9, 6708 WG Wageningen, The Netherlands

## Abstract

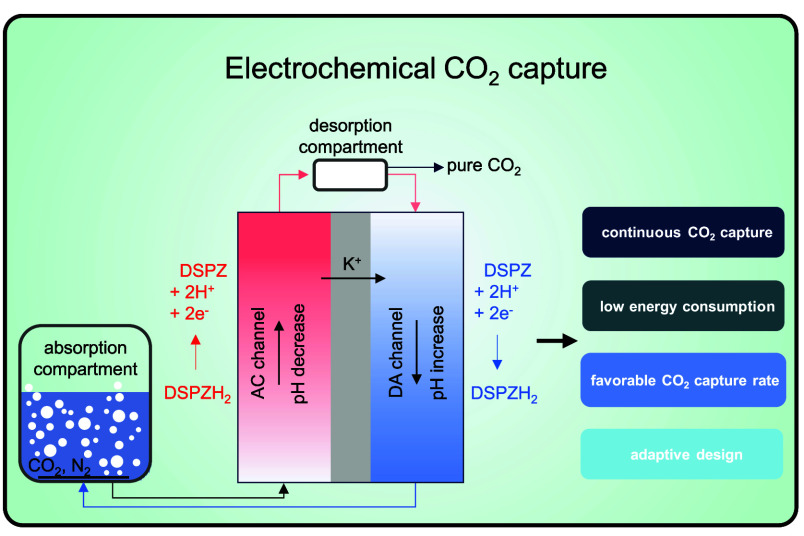

We investigate a continuous electrochemical pH-swing method to capture CO_2_ from a gas phase. The electrochemical cell consists of a single cation-exchange membrane (CEM) and a recirculation of a mixture of salt and phenazine-based redox-active molecules. In the absorption compartment, this solution is saturated by CO_2_ from a mixed gas phase at high pH. In the electrochemical cell, pH is reduced, and CO_2_ is selectively released in a desorption step. We investigate the influence of redox molecule concentration on the charge storage capacity of the solution, as well as the impact of current density and solution recirculation rate on process performance. A theoretical framework, based on a minimal set of assumptions, is established. This framework describes the data very accurately and can be used for system design and optimization. We evaluate the trade-off between energy consumption and CO_2_ capture rate and compare with published reports. We report a low energy consumption of 32 kJ/mol of CO_2_ at a capture rate of 39 mmol/m^2^/min.

To remove, or capture, CO_2_ from a gas phase, electrochemical methods are considered promising as a technology due to their ease of integration with renewable electricity, low energy consumption, and ambient operating conditions.^1−4^ Various electrochemical pH-swing methodologies were developed for CO_2_ capture under ambient temperature and pressure, including bipolar electrodialysis,^5−7^ H_2_-recycling electrochemical systems,^8−10^ electrochemically driven proton concentration processes,^11,12^ and the application of proton-coupled electron transfer reactions (PCET).^13−18^ Other electrochemical methods include the application of capacitive electrodes,^19−21^ the generation of nucleophiles that selectively bind CO_2_ molecules,^22,23^ and electrochemically mediated amine regeneration.^24^ The energy demand for CO_2_ can be further decreased by making use of a renewable power supply.^25^

CO_2_ capture processes based on the pH-swing method can occur either simultaneously in a continuous process or sequentially in a cyclic transport process. We present a method based on simultaneous absorption and desorption of CO_2_, where water containing salt (such as KCl) is continuously recirculated between the absorption and desorption compartments, see Figure 1. In the absorption compartment, a high pH is established, while in the desorption compartment, a low pH is maintained. This difference is caused by the cation-exchange membrane (CEM) placed inside the electrochemical cell that at sufficiently high salt concentration will almost exclusively transport cations (K^+^ in this example). For pH values used in our study, the transport of H^+^ across the membrane is minor. To compensate for the K^+^ flux leaving or entering a compartment, H^+^ ions (shorthand for the hydronium ion, H_3_O^+^) are formed or removed (or, vice versa, OH^–^ ions are removed or formed), which effectively occurs at the electrodes. The simple reactions of H^+^ to H_2_ and water to O_2_ can be used for that, but these reactions are not ideal because of the formation of gas bubbles and the high overpotential which increases energy consumption and can induce parasitic reactions such as the formation of chlorate. To avoid these complications we use a specific type of redox-active molecule that is reduced on the cathode where it reacts with two electrons and two protons. On the anode, these molecules are oxidized and release both the electrons and the protons. Because in both the reduced and oxidized state the molecule has the same (negative) charge, the charge of the DSPZ molecule does not influence the electroneutrality balance but only plays a role in the creation and annihilation of protonic charge. While in electrodialysis, redox molecules can be confined to an “electrode rinse solution” that cycles between the two end-electrodes, in the present process, this recirculation is combined with the primary circulation of the aqueous solution and CO_2_.^26^

**Figure 1:**
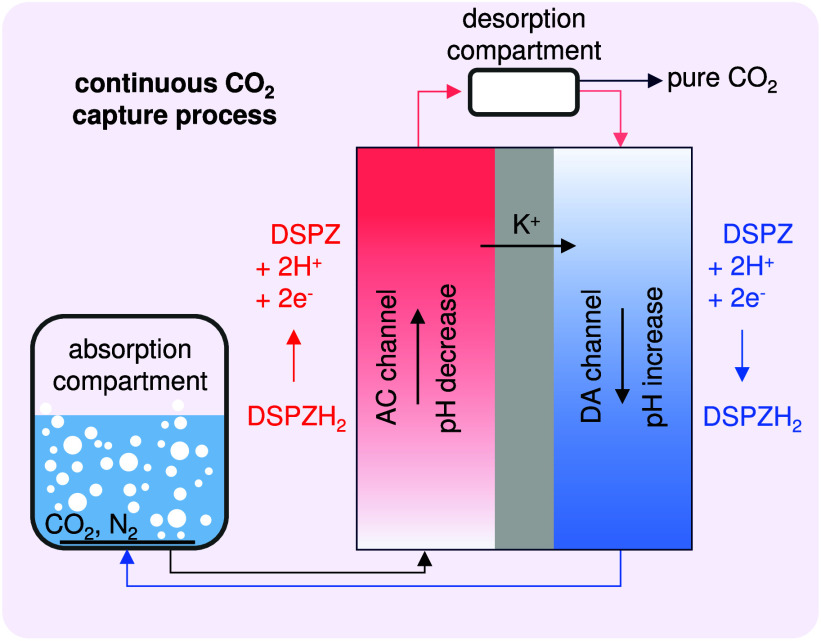
Schematic overview of the continuous flow electrochemical cell. In the absorption compartment on the left, CO_2_ is absorbed at high pH and then flows into the anode, or acidification (AC) channel, where H^+^ is formed (or equivalently, OH^–^ is removed), resulting in a pH decrease and CO_2_ gas removal in the desorption compartment. Subsequently, in the cathode, or deacidification (DA) channel, pH is reduced, the CO_2_ absorption capacity is restored, and the solution returns to the CO_2_ absorption compartment.

In the present paper, we show the very favorable performance of this type of electrochemical design for carbon capture and compare it with reported performance metrics from the literature. In conventional pH-swing electrochemical carbon capture technologies,^10,28^ the energy performance of the CO_2_ capture process is limited by the large pH difference between the acidification and deacidification compartments and the concentration polarization at the membrane–solution interfaces. In our study, we achieved low energy consumption and a relatively high CO_2_ capture rate by using a high concentration of background electrolyte to prevent the development of concentration polarization, along with a phenazine-based redox molecule. This type of organic compound has a low overpotential and is therefore suitable to lower the energy consumption of CO_2_ capture processes.

Phenazines have been studied as active compounds for redox-mediated pH-swing processes.^14−18^ A sodium-phenazine-sulfonate molecule (DSPZ) was synthesized and studied as the redox compound in a cyclic electrochemical process for CO_2_ capture.^15,16^ In the method of refs (15) and (16), DSPZ was used at one electrode and potassium ferrocyanide on the other. In that method, the reduction of one DSPZ molecule results in reaction with two H^+^ ions from solution, resulting in an increase in pH, while in the oxidation step, two H^+^ ions are produced. As in the cyclic process of refs (15) and (16), the reduction and oxidation of DSPZ occur at one electrode, and the oxidation and reduction of other molecules such as potassium ferrocyanide K_4_[Fe(CN)_6_] to potassium ferricyanide K_3_[Fe(CN)_6_] are required at the other electrode. Using two different electrolytes on each side of the cell as proposed in refs (15) and (16) complicates system design and is only suitable for batch operations. This cell design is furthermore disadvantageous, as only part of the total operational time is used for the CO_2_ capture process, and cross-contamination of the catholyte and anolyte increases in time. Another promising process scheme, reported in ref (14), involves batch operation with phenazine molecules used in both the anode and the cathode, while ref (27) presents a second approach based on continuous electrochemical carbon capture using different electrolytes in the anode and cathode. Instead, in the present work we use a method where only DSPZ is cycled between the anode and cathode, avoiding the complexities encountered in refs (15) and (16). Importantly, the proposed design can be combined with the electrochemical rebalancing method described in ref (16) to mitigate side reactions caused by oxygen. In addition, we compared the experimental findings with a theoretical process model that captures the essential physical and chemical phenomena, making only a few simple assumptions. We will discuss differences between theory and data and how the theory can be improved in the future.

Thus, in this study, we explore both experimentally and theoretically a continuous electrochemical CO_2_ capture process. Experimental evaluations were conducted with feed gases containing 10% and 20% CO_2_, examining the system performance for different current densities, solution flow rates, and two cell designs. Furthermore, we compare the measured energy consumption of our system with literature values obtained from other electrochemical methods. Our comparison includes the energy consumption in kJ/mol of CO_2_ removed and the carbon capture rate, which is defined as the amount of CO_2_ captured normalized by the membrane area and operation time. These metrics are essential for evaluating the operational costs of the system.

The system we use for the selective transport of CO_2_ from one gas stream to another consists of a CO_2_ absorption compartment and a desorption compartment, with a single membrane-electrode flow cell placed in between. From the absorption compartment, solution flows into the anode or acidification (AC) channel, from there to the desorption compartment, next back into the cell, to the deacidification (DA) or cathode channel, and then back to the absorption compartment. Current runs between the two electrodes from the anode to cathode, i.e., from the AC channel across the CEM to the DA channel (from left to right in Figure 1). Because of the applied current from the anode to cathode, cations, K^+^, migrate across the CEM, and we assume they have a transport number close to 1 (for each electron, one cation flows across the membrane). The redox-active DSPZ molecule releases two protons and two electrons in the AC channel (anode compartment), after which it flows with the solution to the DA channel (cathode compartment), where it picks up two electrons and two protons. Previously published work has modeled a similar system operating in a cyclic manner.^29^

The theory includes dynamic mass balances for each of the four compartments and for each component. We modeled each of these four compartments as a stirred tank. In the absorption compartment (subscript abs), for instance, we have one such balance for the K^+^ ion, which is
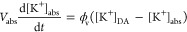
1where *V*_abs_ is the volume of the absorption compartment (mL), *t* is time (min), and ϕ_v_ is the volumetric flow rate by which the solution is recirculated (in mL/min).

In the desorption compartment, there is a similar balance in K^+^, but the inflow is now from the AC channel. The K^+^ balance in the two channels must include transport through the membrane. For the AC channel, it is
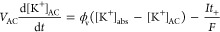
2where *I* is the current between the electrodes (unit A = C/s), *F* is Faraday’s number (96 485 C/mol), and *t*_+_ is the transport number of cations, which for a good membrane is a number close to 1. In this work, we assume *t*_+_ = 1. If we were to use a lower number, we would have to decide how much of the remainder is due to anion transport (Cl^–^) in the opposite direction, what is due to HCO_3_^–^ and what is due to CO_3_^2–^, and what then remains is due to H^+^ and/or OH^–^.

Equation 2 is valid for the AC channel, and we use *I* as a positive number. For the DA channel, we have a similar equation with inflow now from the desorption compartment, and the minus sign in front of *I* is changed to a plus sign.

The other balance is in total inorganic carbon (TIC), which is a summation over all carbonate-containing species, which are H_2_CO_3_, HCO_3_^–^, and CO_3_^2–^. We do not distinguish between absorbed CO_2_ and hydrated H_2_CO_3_, but we use H_2_CO_3_ to describe these two species jointly.

For the absorption compartment, this balance is
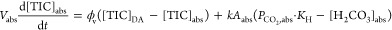
3where [TIC] is the sum of the concentrations of H_2_CO_3_, HCO_3_^–^, and CO_3_^2–^. The transfer coefficient *kA*_abs_ is in mL/min. The partial pressure of CO_2_ is in bar, and the Henry coefficient for CO_2_ absorption is *K*_H_ = 34 mM/bar. The CO_2_ partial pressure is a fixed value based on inlet conditions. We do not include a gas phase CO_2_ balance but assume the CO_2_ concentration here in this gas phase is a known value. We included in eq 3 a mass transport limitation for CO_2_ to diffuse from the gas phase into the solution, described by the mass transfer coefficient *kA*_abs_ in m^3^/s. This value, and thus the overall absorption rate, will depend on how intensely we mix a solution.

For the desorption compartment, the balance is similar, except that the plus sign in front of *kA*_abs_ becomes a minus, we use *kA*_des_, and the CO_2_ partial pressure that is used is an established “backpressure” (*P*_back_ = 0.1 or 0.5 bar in our work) minus the partial pressure of the water that evaporates, which is *P*_water,saturated_ = 0.030 bar; thus *P*_CO_2_,des_ = *P*_back_ – *P*_water,saturated_. For the balance of TIC in the two membrane channels, this is like eq 2, with only accumulation, inflow, and outflow.

In each compartment, several additional equations are solved at each moment in time. First, there is charge neutrality, which is given by
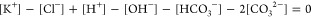
4which applies in each compartment. We do not have to include the redox molecule because its negative charge is constant, and thus in the theory we can simply treat it as if it is part of the Cl^–^ ions. Furthermore, in each compartment we implement [TIC] = [H_2_CO_3_] + [HCO_3_^–^ ] + [CO_3_^2–^].

Finally, in each compartment we must solve the two bicarbonate equilibria, which are
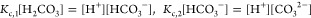
5where *K*_*i*_ has the same unit as [H^+^]. *K* values are derived from the corresponding p*K* value according to p*K* = log_10_*K*, resulting in a *K* that is in mol/L, i.e., M. The two p*K* values we use are p*K*_c,1_ = 6.071 and p*K*_c,2_ = 9.953, based on commercial ion-equilibrium software (Visual Minteq) which includes the effect of salt concentration on p*K*.

The energy consumption in this cell is the electrical power, which is given by current × cell voltage. This energy (unit J/s) can be divided by the CO_2_ capture rate in mol/s to arrive at the energy per mole of CO_2_ removed, see Figures 6 and 7. As shown in Figure 6, in our experiments, the voltage is proportional to the current density, thus the system can be described by a constant resistance *R*, and the electrical power is then *P*_e_ = *I*^2^*R*. In a more detailed theory, the electrochemical reactions of DSPZ inside the porous electrodes that are placed along the membrane are theoretically described (for instance, by the Butler–Volmer equation), as well as ion transport through the electrode and across the membrane based on the Nernst–Planck equation. This description includes all ions individually, including the three states of the bicarbonate ion, as well as H^+^ and OH^–^, see refs (30) and (31). The model must consider the two-dimensional geometry of the flow channel, with ion transport mainly in the direction toward the membrane and water flow along the membrane. Such a detailed model can be used for accurate system optimization.

This finalizes the theory used in our paper. Thus, we only have mass balances, chemical equilibrium, charge neutrality, and a relationship between the cation flux across the membrane and applied current. The only mass transfer relationships are those for CO_2_ absorption and desorption (Figure 2)

**Figure 2:**
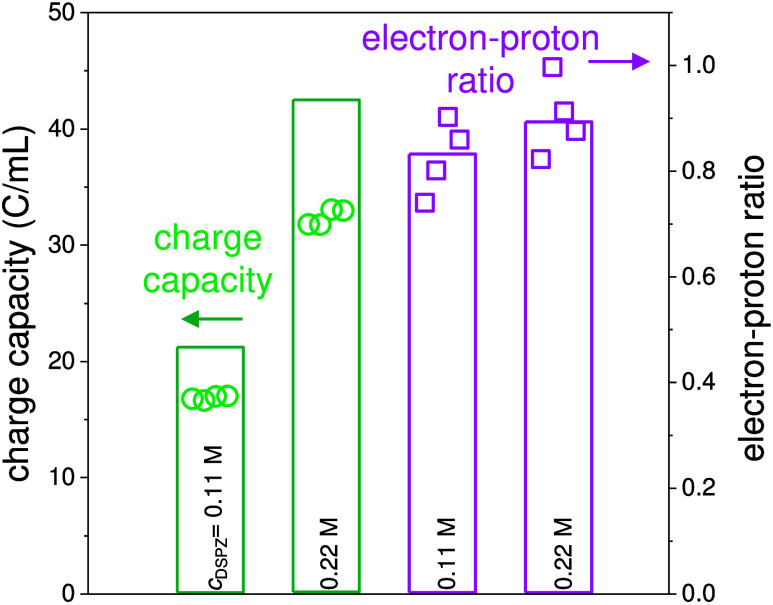
Comparison of the maximum charge capacity assuming 100% purity and complete oxidation/reduction of each DSPZ molecule, plotted as bars, and experimental data of charge capacity based on electrochemical testing (green circles) at two concentrations of DSPZ, namely, 0.11 and 0.22 M. The electron–proton ratio is calculated based on measured charge and pH of the reduced DSPZ solution (pink squares), with median values shown as bars.

We use this model in Figures 3C,D to compare the calculation output with data based on pH that is measured at three positions in the system as well as the measured CO_2_ capture rate. We define the charge capture ratio as the current induced carbon capture rate (in mol/time) divided by the current (in A), multiplied by *F*. The current induced CO_2_ capture rate is the capture rate at a given current minus the baseline capture rate that occurs at zero current. We make this correction both in the experimental analysis and in the theoretical calculations. After each step change in the applied current, it can take a few minutes to reach a steady state, and we only analyze charge capture ratio in this steady state.

**Figure 3:**
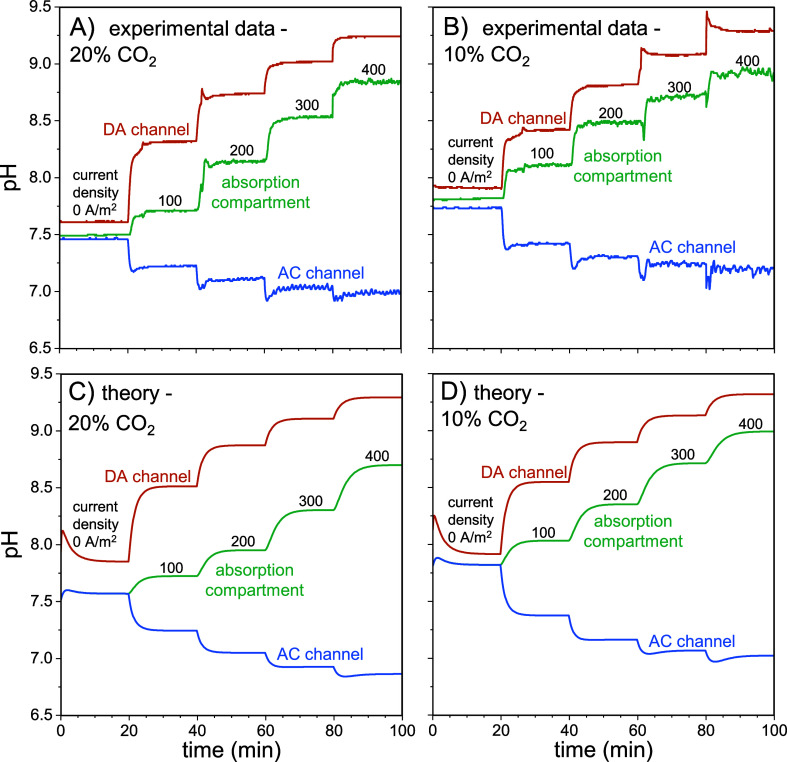
pH recorded as a function of time during continuous CO_2_ capture in the absorption compartment (green line), at the exit of the anode (AC) channel (blue line), and at the exit of the cathode (DA) channel (orange line), with current density increasing stepwise every 20 min for (A) 20% and (B) 10% CO_2_ in the feed gas. (C, D) Model calculations based on our theory that are in very good agreement with the data.

Electrochemical characterization experiments were conducted using 0.11 or 0.22 M DSPZ dissolved in a 1 M KCl solution. The DSPZ solution was first fed into the cathode compartment of the electrochemical cell. In the anode compartment, the solution consisted of a mixture of 0.1 M K_4_Fe(CN)_6_ and 0.1 M K_3_Fe(CN)_6_ in a 1 M KCl solution. When a constant cell voltage of 1.6 V was applied to the electrochemical flow cell, DSPZ was reduced to DSPZH_2_ at the cathode, and Fe^2+^ was oxidized to Fe^3+^ at the anode. When the polarity of the two electrodes was reversed (applied constant cell voltage of −0.5 V), DSPZH_2_ was oxidized back to DSPZ, while Fe^3+^ was reduced to Fe^2+^. We performed four reduction/oxidation cycles of the DSPZ solution, and for an example of one cycle, see Figure S2. Figure 2 presents the theoretical charge capacity of the solution, indicated by the bar level, based on the concentration of DSPZ in solution and assuming that each DSPZ molecule absorbs two electrons and two protons when being reduced, along with the corresponding experimental data points from electrochemical testing; see the Supporting Information (SI) for details.

We observe that the experimental charge capacity (green circles) nearly doubles when the DSPZ concentration is increased from 0.11 to 0.22 M. This experimental capacity is ∼75–80% of the maximum value based on molar mass. This can be due to the synthesized compound not being 100% pure and because under the testing conditions of oxidation/reduction at the cell voltage used, not all DSPZ was fully oxidized/reduced. Figure 2 also displays the electron–proton ratio, calculated using the experimental charge capacity and based on the measured pH of the reduced DSPZ solution (for details, see the SI). The calculated ratio is around unity, which indicates that the reaction of DSPZ consumes an equal number of electrons and protons, or in other words, for each electron transferred, one proton is consumed or formed.

Before the continuous carbon capture experiment, half of the DSPZ is electrochemically reduced to DSPZH_2_ using the same flow cell as that for the electrochemical characterization experiments. Following this pretreatment step, the solution is in the oxidized form for 50% and in the reduced form for 50%. Next, in the absorption compartment, the solution is equilibrated with either 10% or 20% CO_2_ in a mixture with N_2_, mimicking two concentrations of flue gas. This is called the feed gas further on. Due to the high pH of the solution, CO_2_ is absorbed from the gas phase into the solution. Then, the CO_2_-rich solution is fed to the acidification (AC) channel of the cell (anode compartment), after which it enters the desorption compartment and returns to the electrochemical cell in the deacidification (DA) channel (cathode compartment). From there, it goes back to the absorption compartment. The volumes of all compartments and all other parameters used in the model are provided in Table S1 in the SI. The total solution volume was 40 mL, and the default solution circulation rate through the cell is ϕ_v_ = 15 mL/min unless otherwise noted (Figure 5B). The experimental pH change after a stepwise increase in applied current density is shown in Figure 3. As expected, with increasing current, the pH in the AC channel decreases, while the pH in the DA channel increases. Interestingly, we also observe an increasing pH in the absorption compartment with increasing current. This higher pH in the absorption compartment is because the solution is not fully saturated with the CO_2_ from the feed gas. In the continuous process, both with and without current, two CO_2_ gas–liquid exchange processes take place: first, the CO_2_ absorption in the absorption compartment, and second, the CO_2_ desorption in the desorption compartment, see Figure 1. The steady state is reached when the absorption rate and the desorption rate of CO_2_ are equal, which we also call the carbon capture rate. The absorption rate is determined by the CO_2_ mass transfer coefficient describing the transport from the gas to the liquid phase and depends on the concentration difference between the two phases. The desorption rate is determined by pH, the total carbon concentration in solution, the gas phase pressure (“backpressure”), and the gas–liquid mass transfer coefficient.

When the current is increased, the solution pH in the AC channel (anode) decreases, leading to an increased CO_2_ desorption rate in the desorption compartment. In Figure 3A,B, especially at 300 and 400 A/m^2^, sharp changes in pH are observed after a change in current, which might relate to an influence of current on the pH sensor, but this remains to be further investigated. After the desorption compartment, the solution returns via the DA channel (cathode) to the absorption compartment. If the solution in the absorption compartment is at equilibrium with the gas, the pH in the absorption compartment will remain the same as the initial pH. However, we observe an increase in the pH in the absorption compartment, which is caused by a limited CO_2_ absorption rate from the gas phase to the liquid phase. Consequently, the CO_2_ desorption rate will be reduced.

Comparing the results in Figure 3 for 20% and 10% CO_2_ in the feed gas, we notice that for a higher CO_2_ partial pressure in the gas phase, more CO_2_ is absorbed due to the higher driving force. This is observed even without applying a current, as the pH values across all solutions are lower when exposed to a gas phase with 20% CO_2_ compared to 10% CO_2_. The model calculations depicted in Figure 3C,D show very good agreement with the experimental pH data, assuming a CO_2_ mass transfer coefficient of *kA*_abs_ = 45 mL/min for 20% feed gas CO_2_ and *kA*_abs_ = 60 mL/min for 10% feed gas CO_2_. For the desorption chamber, in both cases, we use *kA*_des_ = 90 mL/min.

During the carbon capture experiments, the total amount of CO_2_ was monitored over time, as illustrated in Figure 4. Though the degradation of DSPZ due to the presence of oxygen has been reported in ref (16), we find that over the course of experimental work, the synthesized DSPZ maintained its performance in an oxygen-free environment. Please note that already without an applied current, a pH difference develops between the two sides of the cell, and CO_2_ is effectively transported. The driving force is the reduced pressure that is imposed in the desorption compartment (0.1 bar absolute). After applying an electrical current to the cell, the rate of CO_2_ capture increases because of the reduced pH in the AC channel. Overall, the cumulative amount of CO_2_ increases linearly over time, illustrating steady state operation of the electrochemical cell.

**Figure 4:**
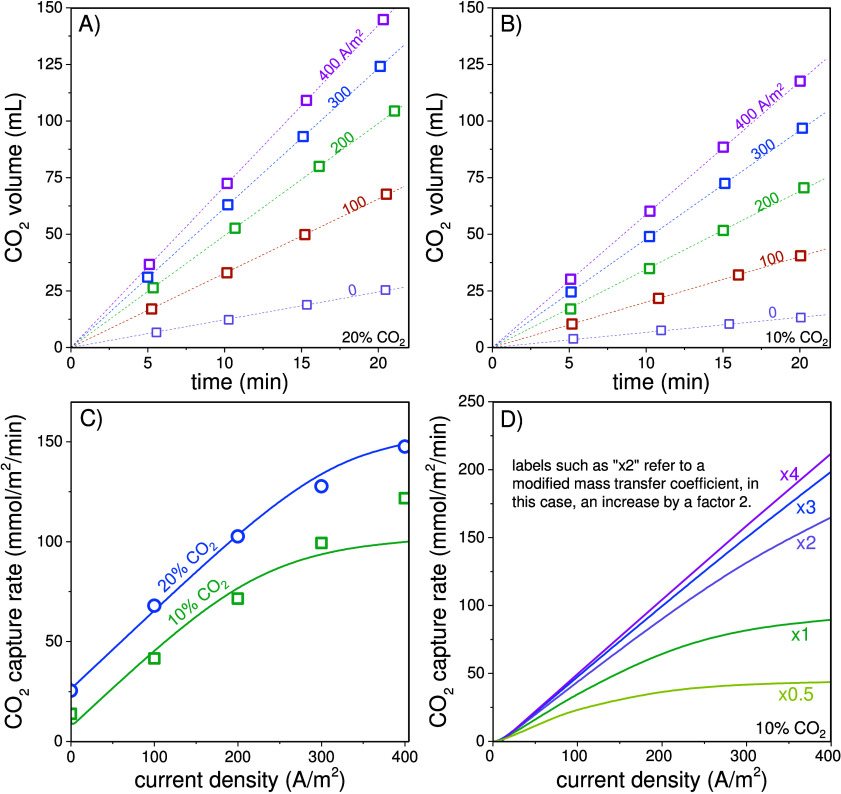
(A, B) Cumulative CO_2_ gas volume captured as a function of time and applied current density for (A) 20% and (B) 10% CO_2_ in the feed gas. Lines are linear fit lines that run through the origin. (C) The carbon capture rate as a function of applied current density and (D) the effect of optimizing the CO_2_ mass transfer coefficient in the absorption and desorption compartments. Labels such as “×2” refer to modified mass transfer coefficients, in this case, an increase by a factor of 2. In (D), the CO_2_ capture rate is adjusted for the capture rate at zero current.

Next, we define the carbon capture rate as the amount of CO_2_ captured per unit of operating time and per projected membrane surface area (20 cm^2^). Figure 4C illustrates the carbon capture rates of the experiments with 20% and 10% CO_2_ in the feed gas. As previously discussed, for the same current density, experiments with a higher CO_2_ partial pressure also have a higher capture rate. The difference increases when the current increases. The lines are based on our theory, and they give a very close fit to the data (parameter settings are the same as for Figure 3). In Figure 4D we theoretically analyze the impact of CO_2_ mass transfer coefficients, *kA*, on the carbon capture rate. The two *kA* values for absorption and desorption of CO_2_ have a large effect on the carbon capture rate, nearly increasing by 3× when mass transfer coefficients are increased 4 ×. The notation along the lines in Figure 4D such as “×2” refers to the increase in both *kA* values, compared to the reference value.

The charge capture ratio of the electrochemical cell is defined as the ratio between the CO_2_ capture rate and current (both expressed in mol/time). As shown in Figure 5A, the charge capture ratio increases slightly when the CO_2_ partial pressure of the feed gas increases. Both experiments and theoretical analyses with 20% CO_2_ indicated a decreasing trend of charge capture ratio with increasing current density. In these experiments the highest charge capture ratio is 69%. The charge capture ratio is also influenced by the solution recirculation flow rate (Figure 5B). These results were obtained in a cell where the electrode compartment thickness was reduced from 1 mm to 500 μm, while the membrane thickness was reduced to a 45 μm thickness. One reason for this is that the recirculation flow rate directly affects the residence time of the solution in both AC and DC channels. With a higher recirculation rate through the AC channel at the same applied current density, the solution is acidified to a lesser extent per pass. As a result, less CO_2_ is degassed in the desorption compartment.

**Figure 5:**
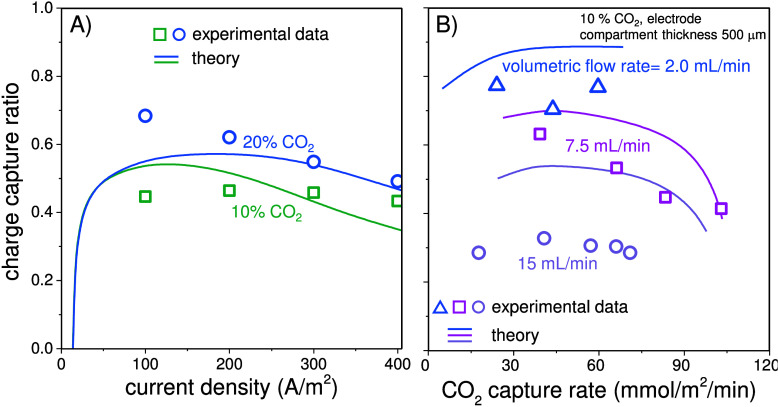
(A) The charge capture ratio, which is the CO_2_ capture rate divided by the current, at 10% and 20% CO_2_ in the feed gas. Solid lines are calculation results based on the model described in this work. (B) Experimental and theoretical capture rate at different recirculation flow rates for 10% CO_2_ in feed gas and reduced electrode compartment thickness (500 μm).

Finally, we discuss the energy consumption of our electrochemical flow cell for carbon capture. Figure 6A shows the change in cell voltage with increasing current density for four experimentally tested conditions. A first observation is that the system we developed has a very low overall resistance. This resistance can be calculated from the slope of voltage versus current density in Figure 6A, and it ranges from 1.9 to 3.2 mΩ m^2^. The resulting low cell voltage must be due to the use of the DSPZ redox molecule as a key component, combined with the high background electrolyte concentration. This characteristic makes the described electrochemical system a promising candidate for carbon capture processes (see also Figure S3). Additionally, it is observed that within the tested range of current densities, the linear increase in cell voltage indicates a constant electrical resistance in the cell.

**Figure 6:**
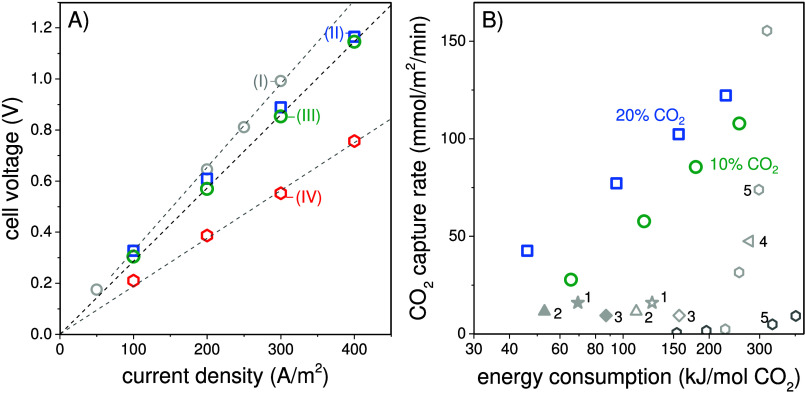
(A) Measured cell voltage as a function of applied current for four different experimental configurations: (I) 10% CO_2_, *c*_DSPZ_ = 0.11 M, Φ = 15 mL/min, δ_mem_ = 130 μm, δ_channel_ = 1 mm; (II) 20% CO_2_, *c*_DSPZ_ = 0.22 M, Φ = 15 mL/min, δ_mem_ = 130 μm, δ_channel_ = 1 mm; (III) 10% CO_2_, *c*_DSPZ_ = 0.22 M, Φ = 15 mL/min, δ_mem_ = 130 μm, δ_channel_ = 1 mm; and (IV) 10% CO_2_, *c*_DSPZ_ = 0.22 M, Φ = 7.5 mL/min, δ_mem_ = 45 μm, δ_channel_ = 0.5 mm. (B) Overview of experimental performance in terms of carbon capture rate in mmol/m^2^/min and energy consumption in kJ/mol of CO_2_ captured. Blue squares and green circles are from the present study, while gray stars, triangles, diamonds, and hexagons are from the literature: 1,^15^ 2,^18^ 3,^16^ 4,^10^ and 5.^32^ See Table S2 for details.

The CO_2_ capture rate and energy consumption are critical for the widespread adoption of a carbon capture process. In Figure 6B we present data for the measured CO_2_ capture rate at 10% and 20% CO_2_ concentrations in the feed gas and the corresponding value of energy consumption, both from our own work and from the literature. The results show that a higher CO_2_ capture rate correlates with an increase in the level of energy consumption. This is mainly due to the larger cell voltage at higher current densities. When comparing different CO_2_ partial pressures in the feed gas, we find that 20% CO_2_ results in a lower energy consumption than 10% CO_2_, indicating the advantage of the proposed system for flue gases that have a higher CO_2_ concentration (additional details for comparison can be found in Table S2 and Figure S4). The experimentally measured CO_2_ capture rates in mmol/m^2^/min reported in our study are higher than the literature values reported for a given energy input. It is important to note that the final capture rate presented in Figure 6B depends on the experimentally achieved CO_2_ mass transfer coefficient during both the absorption and desorption processes. For example, in Figure 6B, values of 16 and 9.5 mmol/m^2^/min (represented by an open star and open diamond) were reported for DSPZ saturated with 47% and 10% CO_2_ feed gas.^15,16^ Another type of phenazine-based redox molecule reached experimental capture rate of 11.5 mmol/m^2^/min (represented by an open triangle).^18^ These studies used a cyclic CO_2_ capture process, resulting in an energy consumption in a range between 157 and 111 kJ/mol of CO_2_. If one would assume 100% energy recovery, then the energy decreases to values between 90 and 53 kJ/mol. In our study, using feed gas with 10% and 20% CO_2_, we achieved an overall energy consumption of 66 and 47 kJ/mol (no energy recovery needed) at higher experimental carbon capture rates of 27 and 42 mmol/m^2^/min, respectively. Thus, continuous operation under the given experimental conditions achieved higher carbon capture rates with competitive energy consumption.

Finally, in Figure 7, we show that the energy consumption can be improved by optimizing process conditions and cell design. To demonstrate that energy reduction is feasible, we decreased the electrode compartment thickness from 1 mm to 500 μm and used a thinner CEM membrane of 45 μm. These cell modifications combined with a lower flow rate of 7.5 mL/min decreased the energy consumption to 32 kJ/mol of CO_2_ removed and attained a carbon dioxide capture rate of ∼40 mmol/m^2^/min (leftmost red hexagon in Figure 7). This result is attributed to the lower resistance in the cell due to a thinner electrode compartment combined with an increased charge capture ratio due to a decreased recirculation flow rate. In Figure 7 we show data from refs (16) and (18) that were based on a cyclic process. We evaluate these data in two ways: first, with and without energy recovery for the actual absorption/desorption condition; second, with and without energy recovery for the situation of high mass transfer coefficients in the absorption and desorption compartments. We then arrive at capture rates of 10–100 mmol/m^2^/min. These results are similar to our own data without optimization of the absorption/desorption mass transfer coefficients.

**Figure 7:**
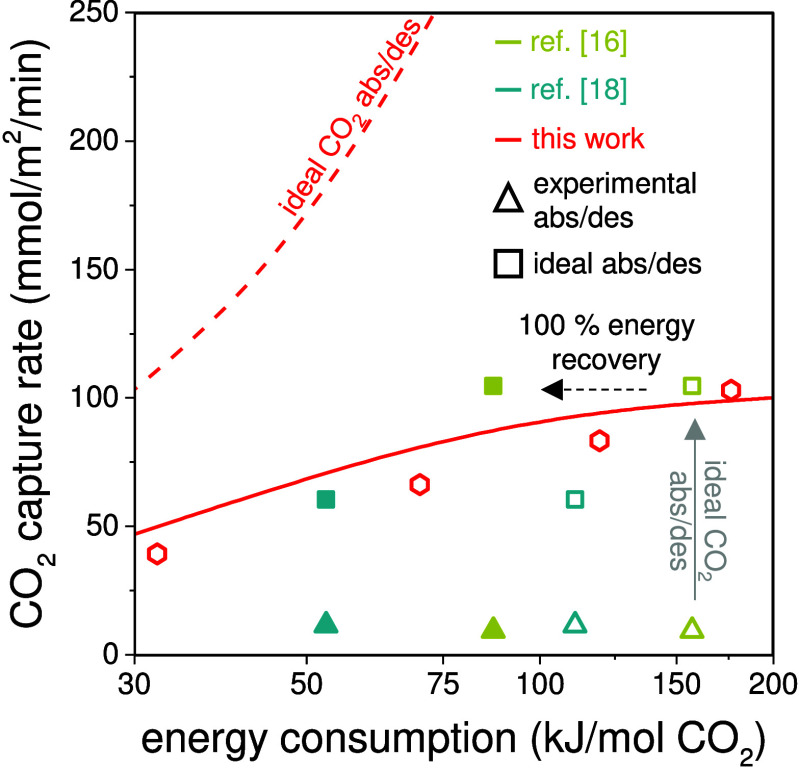
Comparison of performance in terms of carbon capture rate (in mmol/m^2^/min) and energy consumption (in kJ/mol of CO_2_ removed) between the continuous system described in this work (red hexagonal markers) and literature data that were measured in batch-mode systems (all data at 10% CO_2_). Open and closed triangles for the batch system correspond to experimentally measured values without and with energy recovery. Open and closed squares represent values based on ideal absorption and desorption without and with energy recovery. The solid line is based on our model using the same *kA*_abs_ as in Figure 3D, while the dashed line is an ideal scenario with very high mass transfer coefficients. For further details, see Table S2.

The solid red line in Figure 7 is from the theory of this paper with the same parameter settings as in Figure 3D, while we also analyze the ideal case that the absorption and desorption compartments have very high mass transfer coefficients for CO_2_ transport. This is the red dashed line which shows that an improvement in capture by a factor of 2 is feasible at the same energy consumption. This last calculation is further explained in Figure S5, showing the predicted pH profiles with a decrease of pH in the AC channel, while in the absorption compartment pH is now stable in time.

In summary, Figure 7 demonstrates the potential of an electrochemical carbon capture process with phenazine-based redox molecules in a continuous flow cell, achieving a high CO_2_ capture rate with low energy consumption, which showcased the feasibility of large-scale deployment.

Further modifications are necessary to improve the predictions of our model. A notable suboptimal situation is that in the calculations we had to use a different *kA* for the absorption step for the two values of the feed gas composition to be able to fit the data. This indicates that the model does not yet correctly describe the absorption step. Likely, we need a more detailed and precise model for the dynamics of the CO_2_ absorption in the absorption compartment. Additionally, it is important to investigate the transport of ions other than the counterion (either K^+^ or Cl^–^) across the membrane using a multi-ion membrane transport model. Finally, it is important to theoretically study the relation between current and cell voltage and the influence of system dimensions, temperature, and DSPZ concentration (and ratio between reduced and oxidized state).

In conclusion, in this paper, we presented experimental and theoretical results based on the analysis of an electrochemical flow cell designed for continuous carbon capture. This cell uses the same redox solution in both the anode and cathode channels. We analyzed the influence of CO_2_ concentration in the feed gas, applied current, and the solution recirculation rate. Our cell is simple in layout and operates continuously, which is highly advantageous compared with cyclic operation. We show results for energy consumption and carbon capture rate that are as good as or better than results from the literature. We developed a theoretical model that we could validate against experimental data, and it describes the development of pH in all compartments as a function of time and predicts the carbon capture rate. However, mass transfer coefficients for CO_2_ were fitted to data, and a more detailed model is required. In future work, the redox reaction of DSPZ in the porous electrodes must also be described in more detail. In this way, a better design of these electrodes is possible, resulting in a lower overpotential and thus less energy consumption.

## Supporting Information Available

Details of DSPZ synthesis, DSPZ structure, electrochemical characterization methods, CO_2_ capture experiments, electron–proton ratio definition, raw experimental data, energy comparison, effect of the mass transfer coefficient on pH, and tables with parameters and energy comparison values (PDF)
