# Supplementary material for: Continuous Electrochemical Carbon Capture via Redox-Mediated pH Swing—Experimental Performance and Process Modeling
Source: J Phys Chem Lett. 2025 Jan 29;16(5):1343–51. doi: 10.1021/acs.jpclett.4c03111 (PMC11808779; doi:10.1021/acs.jpclett.4c03111)
Supplement: Supplementary file 1 — jz4c03111_si_001.pdf [file jz4c03111_si_001.pdf]

Supporting Information

Continuous electrochemical carbon capture via redox-mediated pH swing – experimental performance and process modeling

P. Śledzik,[1] P.M. Biesheuvel,[2] Q. Shu,[2] H.V.M. Hamelers,[2,3] and S. Porada[1,*]

[1]Department of Process Engineering and Technology of Polymer and Carbon Materials, Wroclaw University of Science and Technology, Wyb. St. Wyspiańskiego 27, 50-370 Wrocław, Poland.
[2]Wetsus, European Centre of Excellence for Sustainable Water Technology, Oostergoweg 9, 8911 MA Leeuwarden, The Netherlands.
[3]Environmental Technology, Wageningen University, Bornse Weilanden 9, 6708 WG Wageningen,The Netherlands.
[*]Corresponding author: Sławomir Porada, slawomir.porada@pwr.edu.pl

1 Experimental

1.1 Synthesis

The synthesis of sodium 3,3'-(phenazine-2,3-diylbis(oxy))bis(propane-1-sulfonate) (DSPZ), a redox molecule, was described previously in [1]. At first, a 0.2 M benzene-1,2-diamine solution was prepared by mixing benzene-1,2-diamine with 2,5- dihydroxycyclohexa-2,5-diene-1,4-dione in water. Subsequently, the mixture underwent reflux at 80 °C until the following day. The resulting slurry was filtered leaving a black precipitate remaining which is crude phenazine-2,3-diol (DHPZ). The product was then dissolved in 0.1 M KOH solution to achieve a 0.02 M DHPZ solution. The filtration process was repeated, and the filtrate was neutralized (pH 7) with an HCl solution and filtered again. As a result, pure red DHPZ was obtained. DHZP was then dissolved in DMF and NaH was added to the solution under a nitrogen atmosphere. When the bubbles stopped emerging from the solution, propane sultone was added and the solution was left to stir overnight at 80 °C. As a result, a red slurry was achieved which was then cooled and filtered. Residual DMF was removed by washing precipitates with ethyl acetate and the final product (DSPZ) was obtained in the form of red crystalline powder.

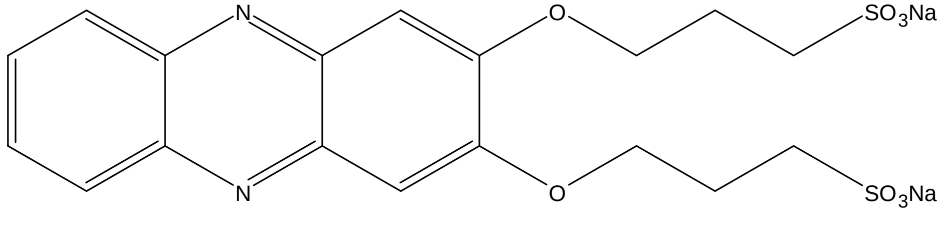

Figure S1: Chemical structure of synthesized redox-active molecule (DSPZ).

1.2 Electrochemical characterization

The experimental setup comprised two electrolyte compartments, a single pH electrode, and an electrochemical flow cell. The electrochemical flow cell was constructed in a sandwich-like configuration, consisting of two symmetrical sides separated by a commercial cation exchange membrane (Neosepta CMX, ASTOM Corporation, Tokyo, Japan) with a thickness of 140 μm. Each side was equipped with a poly(methyl methacrylate) end plate and a graphite current collector. A 1 mm-thick rubber spacer was positioned between the graphite current collector and the cation exchange membrane. The spacer was longitudinally cut to accommodate a 20 cm^2 carbon-felt electrode and to create a flow channel.

Characterization experiments were conducted using two different concentrations of the redox molecule: 0.11 M or 0.22 M of DSPZ in 1 M KCl, and 0.1 M $K_4Fe(CN)_6$ and 0.1 M $K_3Fe(CN)_6$ in 1 M KCl. A pH electrode was used to monitor the pH changes inside the DSPZ electrolyte reservoir. The electrolytes were recirculated between the electrochemical flow cell and the compartments using peristaltic pumps. The DSPZ-containing electrolyte of 40 mL and the $Fe(CN)_6$ electrolyte were recirculated at flow rates of 15 and 40 mL/min, respectively, in a co-current mode. The characterization process was initiated by applying a constant voltage of 1.6 V to the electrochemical flow cell, resulting in an increase in pH from nearly neutral to highly basic due to the reduction of DSPZ. The reduction process continued until the residual electrical current was negligible. Subsequently, the applied voltage was changed to −0.6 V, leading to a decrease in pH to the initial level due to the oxidation of $DSPZH_2$. Throughout the entire process, the DSPZ compartment was maintained under a nitrogen atmosphere to prevent chemical oxidation by oxygen.

1.3 Continuous flow cell operation

To address the challenge of continuous CO_2 extraction using a single electrochemical flow cell with the same type of redox molecules in both the cathode and anode compartments, we have introduced the experimental scheme depicted in Fig. 1 of the main text. Importantly, in this scheme, which utilizes only one electrolyte stream, the outflow from the AC channel (anode) is first directed through the CO_2 desorption compartment, where CO_2 is continuously extracted. Next, it is directed to the DA channel (cathode) to recover CO_2 absorption capacity via electrochemical reduction. The outlet from the DA channel is subsequently directed back to the absorption compartment, where CO_2 from the gas phase is absorbed.

Before initiating continuous CO_2 extraction, the DSPZ solution is charged to half of the total possible charge capacity, as measured during DSPZ characterization measurements. We used the same method as previously described in the DSPZ characterization section to charge the DSPZ-containing solution to half of its maximum charge capacity, measured at a cell voltage of 1.6 V. The half-charged DSPZ solution is then saturated in the absorption compartment with a pre-selected mixture of CO_2/N_2. The saturation step was continued until equilibrium was reached at a given CO_2 partial pressure. Subsequently, a constant current is applied to the electrochemical flow cell. Due to the applied current, the half-charged DSPZ solution flowing through the cell is capable of conducting at the same time both the acidification and deacidification process. After passing through one side of the cell, the electrochemically oxidized DSPZ solution then flows through the desorption compartment maintained at a pressure of 0.1 bar using a peristaltic pump. In the desorption compartment, carbon dioxide is extracted, and then the gas flow is directed through a flow meter to measure the gas volume. The outgassed DSPZ solution is then returned to the other side of the cell, where it is electrochemically reduced (deacidified) again and directed to the absorption compartment where CO_2 is again absorbed from a gas mixture. The amount of

CO_2 captured was measured using a gas flow rate meter connected to the experimental setup. Additionally, three pH electrodes were placed in the experimental setup to monitor the electrolyte pH. One electrode was placed in the absorption compartment, the second electrode was positioned at the exit of the AC channel (before the degassing chamber), and the third electrode was located at the exit of the DA channel (cathode). All continuous CO_2 extraction experiments were conducted using different current densities applied to the electrochemical flow cell and at two different ratios of CO_2/N_2, namely 10 % and 20 %. The total solution flow rate was kept at 15 mL/min unless specified differently.

Additional experiments were conducted with a reduced electrode compartment thickness of 500 μm and a reduced membrane thickness of 75 μm to further optimize CO_2 capture process.

2 Electron-proton ratio

The electron-proton (hydronium ion) ratio of the DSPZ molecule in the oxidation-reduction cycle is calculated as follows. In the most extreme oxidation limit, the pH in the tank is measured to be moderate, indicating low concentrations of H^+ and OH^-. However, in the reduced form, the solution becomes basic with a pH of around 13.0. Therefore, the measured pH in the fully reduced state is an indication of the number of protons absorbed during the reduction of DSPZ. The solution concentration is calculated using Visual MINTEQ software, where we match measured pH to that calculated by the software, and then extract OH^- as the summation of free OH^- and KOH. That value, multiplied by the volume of the solution, gives the amount of H^+ absorbed during reduction process. The ratio is then calculated by considering the measured charge in coulombs, divided by Faraday's constant and the OH^- concentration obtained from Visual MINTEQ, as illustrated in Fig. 2.

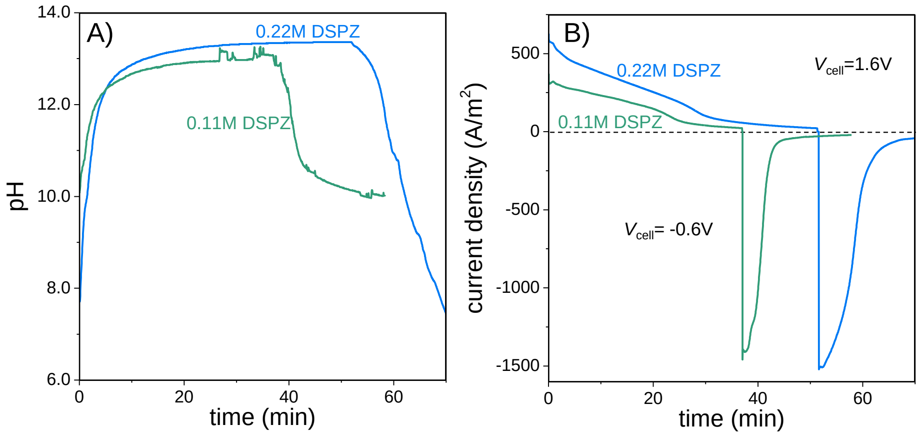

Figure S2: Example cycle of electrochemical characterization of 0.11 M and 0.22 M DSPZ solution in 1 M KCl. A) solution pH changes over time, and B) current density in A/m^2.

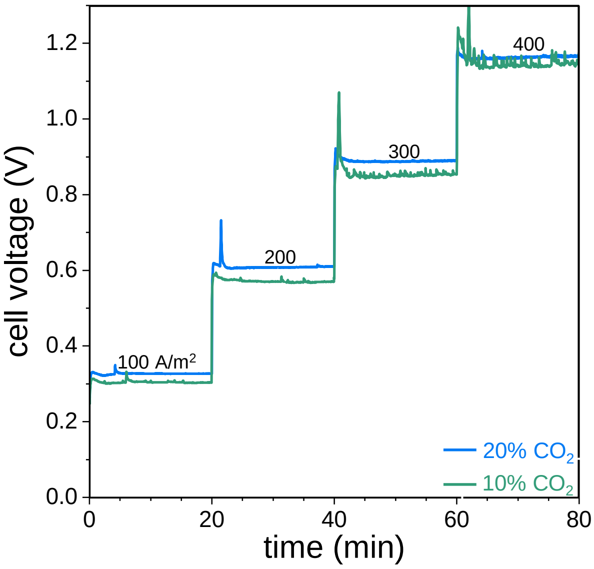

Figure S3: Cell voltage during continuous operation of the electrochemical flow cell with 10 % and 20 % CO_2 in the absorption chamber as a function of applied current density.

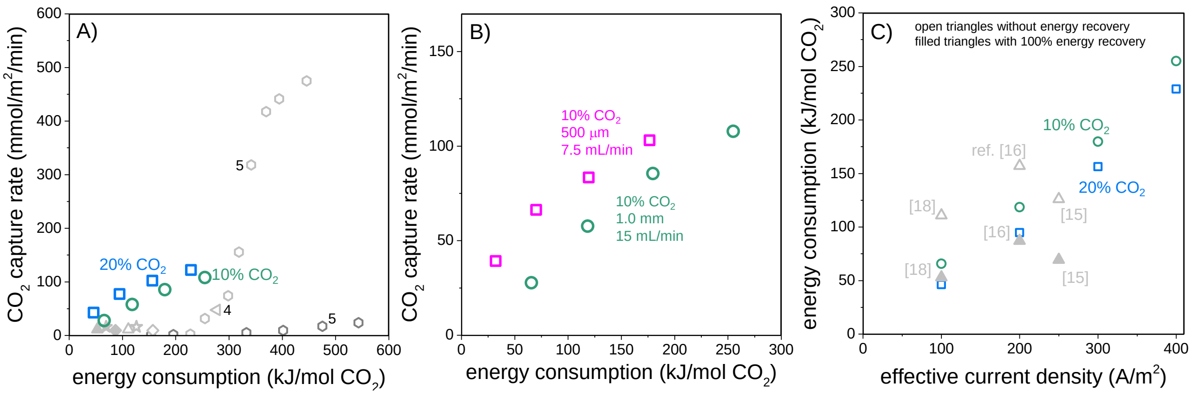

Figure S4: A) Performance comparison in terms of CO_2 capture rate in mmol/m^2/min and energy consumption in kJ/mol of CO_2 removed between measured and literature data. Table S1 below provides more information about the literature data. B) CO_2 capture performance comparison between two electrochemical flow cell designs operated at different conditions. C) Performance comparison in terms of effective current density and energy consumption in kJ/mol of CO_2 removed between measured and literature data.

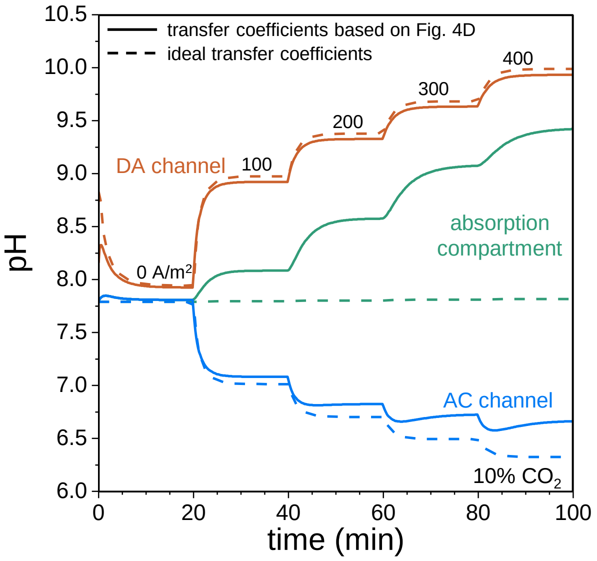

Figure S5: Calculated pH as function of time during continuous CO_2 capture in the absorption compartment (green line), at the exit of the anode (AC) channel (blue line), and at the exit of the cathode (DA) channel (orange line), with current density increasing stepwise every 20 min, for A) mass transfer coefficients as used in Fig. 4D and B) the situation of ideal absorption and desorption (very high mass transfer coefficients).

Table S1: Details of the compartments, flow channel volumes, and other settings for experiments, and theoretical calculations.

total solution volume, V_{tot}	40	mL
absorption compartment volume, V_{abs}	33	mL
desorption compartment volume, V_{des}	5	mL
AC and DA channel volume, V_{AC}, V_{DA}	1, 1	mL
volumetric flow rate, ϕ_{v}	15	mL/min
pressure in the desorption compartment, P_{back}	0.1	bar
$\text{pK}_{\text{c},1}$, $\text{pK}_{\text{c},2}$	6.071, 9.953	-
Henry coefficient for CO_2, K_{H}	34	mM/bar
active cell area	20	cm^2
cation-exchange membrane thickness	140	μm

Table S2: Performance comparison in terms of CO_2 capture rate in mmol/m^2/min and energy consumption in kJ/mol of CO_2 removed between measured and literature data. The values in brackets represent conditions of the ideal transfer coefficients for the CO_2 absorption and desorption processes.

Ref.	operational mode	energy consumption (kJ/mol)	recoverable energy (kJ/mol)	desorption rate (mmol/m^2/min)	partial pressure (-)
1	cyclic	126	57	16	0.465
2	cyclic	111	58	11.5 (60.5)	0.1
3	cyclic	157	70	9.5 (105)	0.1
4	continuous	273	-	48	-
5	continuous	154	-	0.5	-
5	continuous	195	-	1.6	-
5	continuous	332	-	4.8	-
5	continuous	401	-	9.2	-
5	continuous	475	-	17	-
5	continuous	543	-	24	-
5	continuous	629	-	28	-
5	continuous	227	-	2.33	-
5	continuous	255	-	31	-
5	continuous	298	-	74	-
5	continuous	319	-	155	-
5	continuous	342	-	318	-
5	continuous	370	-	420	-
5	continuous	395	-	440	-
5	continuous	445	-	475	-
this work	continuous	32 (19)	-	40 (60)	0.1
this work	continuous	70 (37)	-	66 (122)	0.1
this work	continuous	119 (55)	-	84 (186)	0.1
this work	continuous	176 (73)	-	103 (250)	0.1

Acknowledgments

This work was funded by the National Science Centre, Poland [2022/01/1/ST5/00025] and the Polish National Agency for Academic Exchange [Polish Returns grant BPN/PPO/2021/1/00010]. This work was performed in cooperation with Wetsus,European Centre of Excellence for Sustainable Water Technology (www.wetsus.nl). Wetsus is cofunded by the Dutch Ministry of Economic Affairs and Ministry of Infrastructure and environment, the European Union Regional Development Fund, the Province of Fryslân, and the Northern Netherlands Provinces.

References

[1] S. Jin, M. Wu, R.G. Gordon, M.J. Aziz, and D.G. Kwabi, "pH swing cycle for CO2 capture electrochemically driven through proton-coupled electron transfer," *Energy & Environmental Science* **13**, 3706 (2020).

[2] S. Pang, S. Jin, F. Yang, M. Alberts, L. Li, D. Xi, R.G. Gordon, P. Wang, M.J. Aziz, and Y. Ji,"A phenazine-based high-capacity and high-stability electrochemical CO_2 capture cell with coupled electricity storage," *Nature Energy* **8**, 1126 (2023).

[3] S. Jin, M. Wu, Y. Jing, R.G. Gordon, and M.J. Aziz, "Low energy carbon capture via electrochemically induced pH swing with electrochemical rebalancing," *Nature Communications* **13**, 2140 (2022).

[4] Q. Shu, C.S. Sin, M. Tedesco, H.V.M. Hamelers, and P. Kuntke "Optimization of an electrochemical direct air capture process with decreased CO_2 desorption pressure and addition of background electrolyte," *Chemical Engineering Journal* **470**, 144251 (2023).

[5] P. Zhu, Zhen-Yu Wu, A. Elgazzar, C. Dong, Tae-Ung Wi, Feng-Yang Chen, Y. Xia, Y. Feng, M. Shakouri, J.Y. Kim, Z. Fang, T.A. Hatton, and H. Wang,"Continuous carbon capture in an electrochemical solid-electrolyte reactor," *Nature* **618**, 959 (2023).
